# From Lab-Testing to Web-Testing in Cognitive Research: Who You Test is More Important than how You Test

**DOI:** 10.5334/joc.259

**Published:** 2023-01-19

**Authors:** Kim Uittenhove, Stephanie Jeanneret, Evie Vergauwe

**Affiliations:** 1 University of Lausanne, Switzerland; 2 University of Geneva, Switzerland

**Keywords:** Data quality, Web-testing, Working memory

## Abstract

The transition to *web-testing*, although promising, entails many new concerns. Web-testing is harder to monitor, so researchers need to ensure that the quality of the data collected is comparable to the quality of data typically achieved by *lab-testing*. Our study yields a novel contribution to this issue, by being the first to distinguish between the impact of web-testing and the impact of sourcing individuals from different participant pools, including crowdsourcing platforms. We presented a fairly general working memory task to 196 MTurk participants, 300 Prolific participants, and 255 students from the University of Geneva, allowing for a comparison of data quality across different participant pools. Among university students, 215 were web-tested, and 40 were lab-tested, allowing for a comparison of testing modalities within the same participant pool. Data quality was measured by assessing multiple data characteristics (i.e., reaction time, accuracy, anomalous values) and the presence of two behavioral benchmark effects. Our results revealed that *who you test* (i.e., participant pool) is more important than *how you test* (i.e., testing modality). Concerning *how you test*, our results showed that web-testing incurs a small, yet acceptable loss of data quality compared to lab-testing. Concerning *who you test*, Prolific participants were almost indistinguishable from web-tested students, but MTurk participants differed drastically from the other pools. Our results therefore encourage the use of web-testing in the domain of cognitive psychology, even when using complex paradigms. Nevertheless, these results urge for caution regarding how researchers select web-based participant pools when conducting online research.

## Towards Web-Testing

In recent years, psychology researchers have increasingly transitioned from traditional lab-testing to conducting experiments online on the worldwide web. This entails an important change in the domain of cognitive psychology, which often involves complex experimental paradigms with precise stimulus timings and reaction time (RT) measurements. Therefore, research has traditionally taken place in a lab space with individuals who can easily be reached, such as students or research associates. However, this traditional approach comes with several inherent limitations, such as small samples with an atypical profile (Westernized, Educated, Industrialized, Rich, and Democratic [WEIRD] individuals, Henrich et al., 2010), and a rather slow and costly serial data collection. On the contrary, web-based testing permits the removal of such limitations (e.g., Gagné & Franzen, 2021) by allowing incredibly fast and parallel data collection from a much wider sample than what was previously possible. Today, myriad papers have established the benefits, limits, and best practices of conducting online experiments in different subdomains of human behavioral research in general (e.g., Birnbaum & Reips, 2005; Sheehan, 2018; Mason & Suri, 2012), including cognitive psychology, specifically (e.g., Stewart, Chandler, & Paolacci, 2017; Gagné & Franzen, 2021; Woods et al., 2015; Mathôt & March, 2021).

A first key development to have driven the transition to web-based testing is the impressive accumulation of internet-based technology, which now allows for the hosting of complex experiments online, with reasonably precise timing and RT measurements, which is particularly relevant for cognitive psychology research (e.g., see Grootswagers, 2020; Sauter et al. 2020 for a comprehensive overview of tools and methods, and Anwyl-Irvine et al., 2021 for an overview of timing issues). A second key development to have driven this transition is the rise of dedicated online testing platforms such as MTurk (2005) and Prolific (2014), with the latter specifically geared towards academic researchers (e.g., Palan & Schitter, 2018). These platforms or marketplaces provide the means to (1) recruit research participants from platform-associated pools, (2) select participants with specific characteristics, and (3) reward participants through specialized and secure payment systems. The use of crowdsourcing platforms such as MTurk for data collection in psychology research is ever-increasing. This is evident when using Google Scholar advanced search results, with the keyword ‘psychology’ combined with the phrases ‘mechanical turk’ or ‘prolific academic’. In the years following the creation of MTurk, from 2005 to 2013, a yearly average of 536 psychology papers mentioned this platform. Following the creation of Prolific, from 2014 to 2019, a yearly average of 5,167 psychology papers mentioned ‘mechanical turk’, whereas 196 papers mentioned ‘prolific academic’. More recently, in 2020 and 2021, a yearly average of 13,000 psychology papers mentioned ‘mechanical turk’, and 1,000 mentioned ‘prolific academic’. MTurk clearly remains the most popular platform to date despite the recent increase in the use of Prolific.

In 2020 and 2021, the increase in web-testing and the use of crowdsourcing platforms was exacerbated by the Covid-19 pandemic, since many researchers were suddenly prevented from conducting human behavioral research in the lab, and the only recourse was to move experiments online. Such a sudden and massive transition to experimental testing online is accompanied by an urgent need to assess the quality of data collected online. Specifically, researchers may want to gauge how these data compare to data gathered through lab-testing: are the characteristics of the data comparable (e.g., distribution of values), or are benchmark effects consistently present in these data? The issue of data quality is of particular concern in cognitive psychology, where researchers use complex experimental paradigms and require precise timing and RT measurements. The current study contributes to this issue by disentangling how data quality from web-testing is influenced by two distinct factors: 1) *testing modality*, i.e., the impact of conducting unmonitored web-testing as opposed to monitored lab-testing, and 2) *participant pool*, i.e., the impact of using different web-based pools of participants from varying sources. To this day, current research in either cognitive psychology or the broader field of psychology has not yet examined the distinct impact these factors may have on research data quality.

## Testing Modalities, Participant Pools, and Data Quality

The transition to web-testing is accompanied by concerns about the quality of the data, specifically whether such data are comparable to the data acquired by lab-testing in terms of characteristics, and whether they allow for the replication of findings from the literature (e.g., Gagné & Franzen, 2021; Crump et al. 2013). First, changing *testing modality* from lab-testing to web-testing may have an impact on data quality. Compared to web-testing, lab-testing typically involves a) verification of the participant via in-person contact, b) consistent configuration of equipment (e.g., hardware and software), c) consistent environment, d) facilitated monitoring (e.g., experimenter presence, audio or video recording), and e) facilitated control (e.g., experimenter intervention). On the contrary, for web-testing, it is more difficult to verify whom is performing the experiment (e.g., risk of ‘bots’ or fraudulent participants; Mason & Suri, 2012; Moss & Litman, 2018; Moss et al., 2021). In addition, it is also more difficult to control which hardware (e.g., processor, graphics card) and software (e.g., browser and operating system) is used for completing the experiment. Therefore, timing accuracy during web-testing varies more due to variations in the hardware and software used by different individuals (e.g., Gagné & Franzen, 2021, see also Anwyl-Irvine et al., 2021; Reimers & Stewart, 2015). Moreover, web-tested participants may be more easily distracted from the task, they may be cheating while performing the task, or may be multitasking during the experiment, and experimenters have no knowledge of the testing environment (e.g., Hauser, Paolacci & Chandler, 2019). It is theoretically possible to monitor and intervene remotely during web-testing, yet this is more challenging to implement, especially if one wants to maintain the benefit of rapid and parallel data collection.

Second, data quality may be impacted by the *participant pool*. Much lab-testing is conducted with students, whereas web-based crowdsourcing platforms such as MTurk and Prolific have their own participant pools, which may vary in demographic composition, such as gender, education, age, socioeconomic status, and nationality. These factors can influence the setup in which the participant completes the experiment (e.g., suitable equipment, quiet environment). Participant pools may also differ in their motivation for experiment participation (e.g., monetary reimbursement, course credit, intrinsic interest), their general and specific skills (i.e., language or numerical ability, domain knowledge), and the level of honesty and accountability, which may vary due to different ways of managing participants on the platforms. Participants on crowdsourcing platforms also evolve over time as their individual experience with the associated platform grows. Some individuals even become full-fledged professionals, treating experiment participation as a full-time job (e.g., Sheehan, 2018; Moss et al., 2020). We have included a table in Supplemental File 1 to present a rudimentary idea of the composition of MTurk and Prolific participant pools, and of potential differences that may exist between both platforms.

To summarize, online data quality may be influenced by at least two distinct factors. First, *testing modality*, i.e., the impact of conducting web-testing as opposed to lab-testing, resulting in reduced experimental control and monitoring, yet increased variability in equipment setup and testing conditions. Second, *participant pool*, i.e., the impact of recruiting participants from different pools with varying composition and characteristics. Even though, to our knowledge, no study has examined the separate contribution of both factors to data quality, several studies in cognitive psychology have broadly investigated the quality of data collected from web-testing, as we describe in the following section.

## The Quality of Web-Based Data Collection in Cognitive Psychology

Among the cognitive psychology studies that evaluated the quality of data from web-testing, we specifically focus on studies including RT measurements. Assessing RT is common practice in cognitive psychology, but adds some complications for web-testing. Table 1 provides an overview of these studies, which a) compared data from web-tested crowdsourced participants to data from the lab or the literature, and b) looked at aspects of data quality, such as data characteristics (i.e., mean values, distributions) and the presence of benchmark effects. The studies in Table 1 are consistent in arguing that web-tested crowdsourced participants yield acceptable-to-good data quality and that benchmark effects are generally present in web-tested crowdsourced data. Even though the results in Table 1 are reassuring concerning the use of web-collected crowdsourced data in cognitive psychology, more recent reports from the more general domain of psychology show an increase in issues with reliability, honesty, comprehension, and attention when collecting survey data from MTurk (Peer et al., 2021), echoing several other recent reports (Chmielewski & Kucker, 2020; Moss et al., 2021; Kennedy et al., 2020). Therefore, in the current study, we re-examined the issue of data quality with the two largest crowdsourcing platforms existing to date, Prolific and MTurk. Moreover, we aimed to disentangle the effects of testing modality and participant pool in order to assess their potential distinct impacts on data quality. More precisely, we compared web-tested students to lab-tested students to evaluate the testing modality effect, and web-tested students to web-tested crowdsourced participants to evaluate the participant pool effect. Given that both factors have almost always been confounded, they have yet to be evaluated independently. For example, in the studies reported in Table 1, data from web-tested crowdsourced participants are compared to either data from the literature or from the lab. Therefore, in most studies, both the participant pool (i.e., most literature or lab data were typically obtained from students, research associates, or otherwise accessible populations) and the testing modality (i.e., web-testing vs. lab-testing) are different.

**Table 1:** Overview of Cognitive Psychology Studies Comparing MTurk or Prolific Data to Students or the Literature.


CROWDSOURCE VS. LITERATURE	MAIN OUTCOME

Barnhoorn, Haasnoot, Bocanegra, Steenbergen, 2014	Successful replication of effects from the domain of experimental psychology on MTurk: Stroop, attentional blink, masked priming.

Bui, Myerson & Hale, 2015	Successful replication of effects from the domain of cognitive aging on MTurk: age-related decline in processing speed, effect of practice on age differences, steeper decline in visuospatial processing, mediation of the link between age and working memory by processing speed.

Crump, McDonnell, & Gureckis, 2013	Successful replication of effects from the domain of experimental psychology on MTurk: Stroop, Switching, Flanker, Simon, Posner Cuing, attentional blink, subliminal priming, and category learning.

Kochari, 2019	Successful replication of effects from the domain of numerical cognition on Prolific: distance effect, congruity effect, priming effect.

Simcox & Fiez, 2014	Successful replication of effects from the domain of experimental psychology on MTurk: Flanker, lexical decision.

Yang & Krajbich, 2021	Successful replication of the effect of gaze duration in decision-making using an eye-tracking paradigm on MTurk.

Zwaan & Pecher, 2012	Partial replication of mental simulation effects in language comprehension on MTurk: orientation match, shape match, color match.

**CROWDSOURCE VS. LAB DATA**	**OUTCOME**

Armitage & Eerola, 2020	Testing an experimental effect in domain of musical cognition in the lab and on MTurk: music valence priming. The effect was consistently present and data characteristics were similar between MTurk and the lab.

Pauszek, Sztybel, & Gibson, 2017	Successful replication of benchmark effects from the spatial cueing paradigm on MTurk: left/right advantage, cue type effect, cued axis effect, and cued endpoint effect.

Lumsden, Skinner, Woods, Lawrence, & Munafò, 2016	Testing a Go-No-Go task in the lab and on MTurk. Reaction times were longer for MTurk, and accuracy was lower, but data quality remained acceptable.


## The Present Study

In the present study, we aimed to disentangle the effects of testing modality and participant pool on data quality in cognitive psychology research. Our study explored data quality between testing modalities and participant pools without specifying predictions, but one could reasonably expect lab-tested data to be of higher quality due to higher levels of control and monitoring. To evaluate any testing modality effect while keeping the participant pool constant, we recruited a student sample (i.e., undergraduates) and compared testing modalities within this sample. More precisely, we compared data from monitored lab-tested students to unmonitored web-tested students. To evaluate any participant pool effect while keeping testing modality constant, we compared data from web-tested students to web-tested crowdsourced participants from MTurk and Prolific.

Across testing modalities and participant pools, we implemented an experimental paradigm that assesses one of the most fundamental aspects of cognitive psychology: working memory. Working memory can be understood as the set of processes that support the innate human ability to mentally retain several distinct pieces of information quasi-simultaneously. As such, many human limitations can be understood in terms of how many pieces of information can be actively retained at the same time. This number seems to be around four (Cowan, 2001), and constrains human abilities in every field of human cognition, such as reasoning, language, arithmetic, problem solving, and decision-making (e.g., Daneman & Carpenter, 1980; Ormrod & Cochran, 1988; Barrouillet & Lecas, 1999; Süß et al., 2002; Engle, Kane, & Tuholski, 1999; Wilhelm & Oberauer, 2006). Our paradigm consisted of a fairly general working memory task requiring participants to maintain short series of letters for a short period of time. We evaluated data quality at the individual level by examining data patterns for each participant. We defined a data pattern as the full set of response data that was collected for a single participant. More precisely, we established, for each participant, A) whether their data pattern was anomalous compared to the distribution of all patterns, and B) whether their data pattern reflected the working memory benchmark effects (e.g., Oberauer et al., 2018) expected within our paradigm. The absence of anomalous data patterns and the replication of well-established benchmark effects are important criteria of data quality for many researchers. The proportion of participants with non-anomalous data patterns and whose data reflected the expected benchmark effects seemed to be reasonable metrics to evaluate the effects of participant pool and testing modality on data quality.

## Methods

We varied testing modalities and participant pools while presenting participants with a fairly general working memory task developed in the context of our research.

### Testing Modalities

There were two testing modalities, web-testing and lab-testing. Lab-testing involved: in-person contact at the beginning and at the end of the experiment, verification of the participant’s identity, presence of the experimenter in an adjacent room during the experiment, consistent equipment and environment, and audio recording of participants^1^ during the task with their consent. This lab-testing environment can be considered highly monitored, and therefore was expected to maximize participants’ efforts to comply with instructions for the entire task duration. Web-testing, on the other hand, was not monitored; we only checked Internet Protocol (IP) addresses to make sure participants would not partake in the experiment multiple times. Finally, we also ensured that participants used a laptop or desktop instead of a mobile device for completing the task.

### Participant Pools

As part of a large-scale recruitment effort to collect data with a web-based working memory paradigm (see Author Note), we collected data from 3 participant pools: MTurk, Prolific, and the community of university students (web-tested or lab-tested). We ended data collection via MTurk earlier than for the other pools because preliminary analysis revealed substantial quality issues. For the other pools, we recruited as many participants as possible; sample size was limited only by the financial and time constraints associated with each pool.

The MTurk sample consisted of 196 participants (screened by approval rating >95%, completed 100+ Human Intelligence Tasks [HITs]), following a strongly recommended practice advocated by most methods papers (e.g., Chandler et al., 2014; Peer et al. 2014). The Prolific sample consisted of 300 participants. The Prolific sample was not pre-screened since recent studies indicate that it is currently not strictly necessary on Prolific (e.g., Peer et al., 2021). MTurk and Prolific participants received a monetary reward of approximately 10 USD for an experiment lasting 30–45 minutes (i.e., 15–20 USD/hour). No additional selection criteria were applied to crowdsourced participants (e.g., nationality, age, education) so as to not intentionally recreate WEIRD samples, which are precisely one of the issues in traditional lab-based research. Web-tested students consisted of 215 undergraduate psychology students at the University of Geneva, who participated in exchange for course credit. Lab-tested students consisted of 40 undergraduate psychology students at the University of Geneva, who participated in exchange for a monetary reward of 15 CHF (i.e., 25–32 USD/hour, for an experiment lasting 30–45 minutes, including additional transportation time), or in exchange for course credit. There were no exclusion criteria for either web- or lab-tested students. All participants provided informed consent before starting the experiment.

### Experimental Paradigm

We developed a working memory paradigm that is representative of the domain, and we instructed participants to maintain information in a particular way. Our working memory task can be considered fairly general in the sense that it encompasses many different aspects comprised in typical working memory paradigms (e.g., serial position, output order, memory stimuli, distractor or processing stimuli, several local recognition output probes). The design of our task, coupled with the task instructions, are expected to create the ideal conditions for measuring two specific working memory benchmark effects (Oberauer et al., 2018). Moreover, we developed our paradigm with the aim to be convenient for web-testing, by requiring participants to press one of two keys to respond. Finally, we collected RTs and accuracy scores, which are typically collected for many working memory tasks. As explained in the introduction, collecting RTs and replicating RT benchmark effects pose a particular challenge with web-testing in cognitive science research.

**Figure 1:**
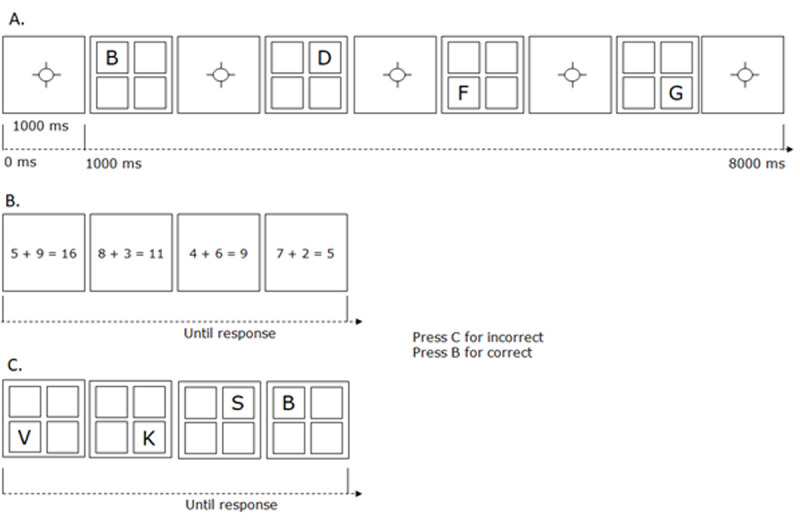
Schematic illustration of the experimental paradigm. *Note*: Panel A shows the presentation phase of memory items. Panel B shows the processing phase. Panel C shows the memory testing phase. Responses to the processing and test items were provided by pressing the B-key with the right index finger for either a correct problem or test item, and the C-key with the left index finger for either an incorrect problem or test item. Of the processing episodes, 50% corresponded to a correct problem and 50% corresponded to an incorrect problem. Each of the four grid positions was probed at the end of each trial, 48.2% of these probes were items from the to-be-remembered list.

The ***working memory task*** (see Figure 1) was composed of a primary memory task (Figure 1, panels A and C) and a secondary processing task (Figure 1, panel B), is often referred to as a Brown-Peterson task, and is frequently used in working memory research (e.g., Oberauer et al., 2018). The memory task consisted of four letters presented sequentially on screen (see Figure 1, panel A). Each letter was presented in one of four spatially distributed boxes on the screen. The secondary task was performed during the memory retention interval (see Figure 1, panel B), and required verification of four arithmetic problems (e.g., 5 + 9 = 16; correct answer is ‘no’). Responses were given by pressing the B-key with the right index finger for a correct problem, and the C-key with the left index finger for an incorrect problem. In the final part of a trial (see Figure 1, panel C), memory for the four letters was tested by sequentially presenting test letters in each of the boxes and asking participants to judge whether each letter corresponded to the letter presented in that box during the presentation of the memory items (B-key for yes, C-key for no). There were 70 trials in total. Each trial recorded 8 responses (4 arithmetic problems and 4 letter memory tests) with 2 response values each (RT and accuracy).

The experiment was hosted on the servers of the University of Geneva and is accessible online via the following link (https://workthatmemory.unige.ch/mturk/), regardless of testing modality. One session of the task took around 30-45 minutes to complete. Responses and RTs were logged via the keyboard. Stimulus presentation times and responses were in the order of 1000 ms; these relatively long durations should not have posed consequential timing precision issues with modern browsers and operating systems (Anwyl-Irvine et al., 2021).

At the beginning of the experiment, we presented a detailed ***instructions tutorial and performance screening***. The different parts of the experiment were explained by providing instructions and practice trials. Participants’ response accuracy was tracked during the practice trials. When accuracy fell below the criterion of 75%, the training was repeated, and continued to be repeated until accuracy reached the criterion, or until the participant abandoned the task. This method ensured that most participants understood and were able to adequately perform the task before beginning the experiment.

We instructed participants to maintain the information by using ***sequential verbal rehearsal***. More precisely, our instructions asked participants to verbally repeat all the letters in their original sequence, and to do so continuously throughout the retention interval in between the letter presentation and letter memory test (e.g., repeat ‘B, D, F, G’ in the example shown in Figure 1). This is a classic rehearsal strategy and is ubiquitous in working memory (e.g., Baddeley, 1986; Barrouillet et al., 2021; Camos et al., 2009; Camos & Barrouillet, 2014; Tan & Ward, 2008).

We tested two working memory benchmark effects that should have been clearly present with this paradigm if participants followed the sequential verbal rehearsal instructions. The first effect pertained to response accuracy and was defined as the ***verbal disruption effect*** (see Oberauer et al., 2018, benchmark 5.2.1). Specifically, the 10 last trials in our task corresponded to verbal disruption trials. These trials involved a significant change in task instructions, and they were presented at the end of the experiment for every participant. We presented these trials in a single block so as to avoid the need to alternate between instructions, and to avoid having these instructions interfere with the implementation of the main instructions in any way. The verbal disruption trials had the same structure as the regular trials, with the sole exception that they required participants to continuously utter ‘mamma mia’ instead of verbally rehearsing the to-be-memorized letters. Uttering such task-irrelevant syllables has been shown to act as a disruption that influences memory performance, especially when the to-be maintained information is verbal in nature (Oberauer et al., 2018). This effect is extremely well-established in the literature (i.e., the articulatory suppression effect; e.g., Baddeley, Lewis, & Vallar, 1984; Camos et al., 2009, Bhatarah et al., 2009; Larsen & Baddeley, 2003; Meiser & Klauer, 1999). Note that in regular trials, the sequential verbal rehearsal strategy should have led to high performance with sets of four letters, which are well within the limits of the articulatory system (e.g., Barrouillet, Gorin, & Camos, 2021). When the use of this strategy is replaced by uttering task-irrelevant syllables in the verbal disruption trials, participants should remember less items or remember them less well, thus producing lower accuracy in the verbal disruption trials. A supplementary analysis ensured that our task did not engender a potential fatigue effect that could be confounded with the verbal disruption effect tested at the end of the task (see Supplemental File 2). We expected the verbal disruption effect to be ubiquitous among participants who complied with the rehearsal instructions. Thus, any absence of this effect could be regarded as a measure of non-compliance. The second benchmark effect was only tested when this first benchmark effect was present.

The second effect pertained to RTs and was defined as the ***RT rehearsal primacy effect*** (see Oberauer et al., 2018, benchmark 3.3.). In our paradigm, the local recognition test required participants to match the position of the letter in each probe to the letter in the corresponding position in the verbally rehearsed sequence. This should have been faster for the first-presented letter, which was also the first letter of the verbally rehearsed sequence. If the first letter of the verbally rehearsed sequence matches the probe letter, participants should have been faster than if they had to move to the next letter in the sequence in order to establish whether it matches the probe. This led us to expect a marked primacy effect (i.e., RT and accuracy benefit for the first-presented letter compared to other letters in the series, e.g., Capitani et al., 1992; Fischler et al., 1970; Oberauer et al., 2018, Palmer & Ornstein, 1971).

Comparing the presence of (1) the verbal disruption effect and (2) the RT rehearsal primacy effect across participant pools and testing modalities informs us whether participants in a given participant pool or testing modality exhibit behavioral patterns that are consistent with those working memory benchmark effects that can reasonably be expected within our paradigm. Moreover, we further examined data quality by examining the presence of anomalous data patterns across participant pools and testing modalities. In addition, we would like to point out that the data are publicly shared, and thus other researchers will be able to evaluate data quality by any metric or benchmark effect that they deem appropriate.

### Data Analysis Plan

We structured our analysis in three main parts, each part dedicated to one of our criteria, and one supplementary analysis. Our point of reference throughout the three main parts are the lab-tested student data, as these data were collected in a highly monitored and consistent setting. For each part, we compared the proportion of data patterns that failed to meet the corresponding criterion, across testing modalities and participant pools, by Fisher exact tests, which are suitable for unequal sample sizes including smaller samples.

#### Part 1: Anomalous data patterns

We compared how many data patterns (i.e., one data pattern corresponds to a complete response dataset from one participant) were anomalous for each participant pool. First, we identified extreme values *within* each data pattern as those RT values which were either extremely small or extremely large. We defined extremely small values as those values that were shorter than 400 ms (see Supplemental File 2 for a detailed explanation). We defined extremely large values within a data pattern as values that exceeded the median RT (Q2) + 1.5 times the interquartile range (IR: Q3-Q1) for that data pattern. Extremely large values may be indicative of technical issues, but may also reflect participant distraction during a given trial. Second, we identified whether a data pattern was anomalous by using a clustering algorithm (see Supplemental File 3 for an explanation of this method) and evaluating whether the data pattern deviated from the other samples in terms of a) the number of extremely small RT values, b) the number of extremely large RT values, c) overall median RT, or d) overall median accuracy. Overall low accuracy may reflect a lack of investment or low skill, whereas overall long RTs may reflect forms of cheating, such as the use of an external device for accomplishing the task.

#### Part 2: Verbal disruption benchmark

Next, we evaluated whether the ***verbal disruption*** benchmark effect was present. Data patterns that showed lower mean accuracy values for verbal disruption trials than for regular memory trials were consistent with the verbal disruption benchmark effect (i.e., worse performance when participants needed to repeat ‘mamma mia’ throughout the retention interval). We did not specify how much lower we expected accuracy to be, and therefore it can be argued that we were conservative in determining when the verbal disruption effect was *not* present. Prior to conducting this analysis, we removed the anomalous data patterns detected in Part 1 of the analysis.

#### Part 3: RT rehearsal primacy benchmark

Third, we compared data quality by evaluating whether the RT ***rehearsal primacy*** effect was present. Primacy-consistent patterns were defined as those data patterns which showed a lower median RT for the first presented letter compared to the average median RT for subsequent letters. These median RTs were calculated for regular memory trials presenting a probe that was indeed present in the memory list, and where the participant gave the correct response. Prior to conducting this analysis, we removed the data patterns that were not indicative of a verbal disruption benchmark effect as detected in Part 2, as the absence of this effect could be regarded as evidence for non-compliance with task instructions.

##### Supplementary analysis

Finally, we retrieved participant characteristics from Prolific participants, and explored whether any specific participant characteristics were linked to data quality. The description and results of this supplementary analysis can be found in Supplemental File 4.

## Results

Table 2 provides an overview of the results following each step of criterion evaluation across testing modalities and participant pools.

**Table 2:** Overview of the proportion of data patterns that do not meet our criteria for each participant pool.


TESTING MODALITY	LAB-TESTED		WEB-TESTED				
	
PARTICIPANT POOL	STUDENTS	FISHER	STUDENTS	FISHER	PROLIFIC	FISHER	

Step 1 N	40		215		300		196

% anomalous samples	7.5		8.4		9.3		17.3

95% CI	[2.6, 19.9]	≈	[5.4, 12.8]	≈	[6.5, 13.2]	*p* < .05	[12.7, 23.3]

Step 2 N	37		197		272		162

% no verbal disruption effect	0		16.2		16.9		42

95% CI	[0.0, 9.4]	*p* < .05	[11.7, 22.0]	≈	[12.9, 21.8]	*p* < .001	[34.6, 50.0]

Step 3 N	37		165		226		94

No rehearsal primacy effect	5.4		5.5		5.3		27.7

95% CI	[1.5, 17.7]	≈	[2.9, 10.0]	≈	[3.1, 9.1]	*p* < .001	[19.6, 37.4]

Final N	35		156		214		68

% of total remaining	87.6%		72.6%		71.3%		34.7%

95% CI	[73.9, 94.5]	≈	[66.2, 78.1]	≈	[66.0, 76.2]	*p* < .001	[28.4, 41.6]


*Note*: The comparisons between participant pools consisted of Fisher exact tests. For each criterion and for each participant pool, we indicated how many participants remained in the sample on which the criterion was evaluated. For each sample, we indicated what percentage did not meet the criterion. For each percentage of data patterns that did not meet the criterion, we presented the 95% Wilson confidence interval, suitable for binomial data and small samples.

### 1. Anomalous data patterns

**Figure 2:**
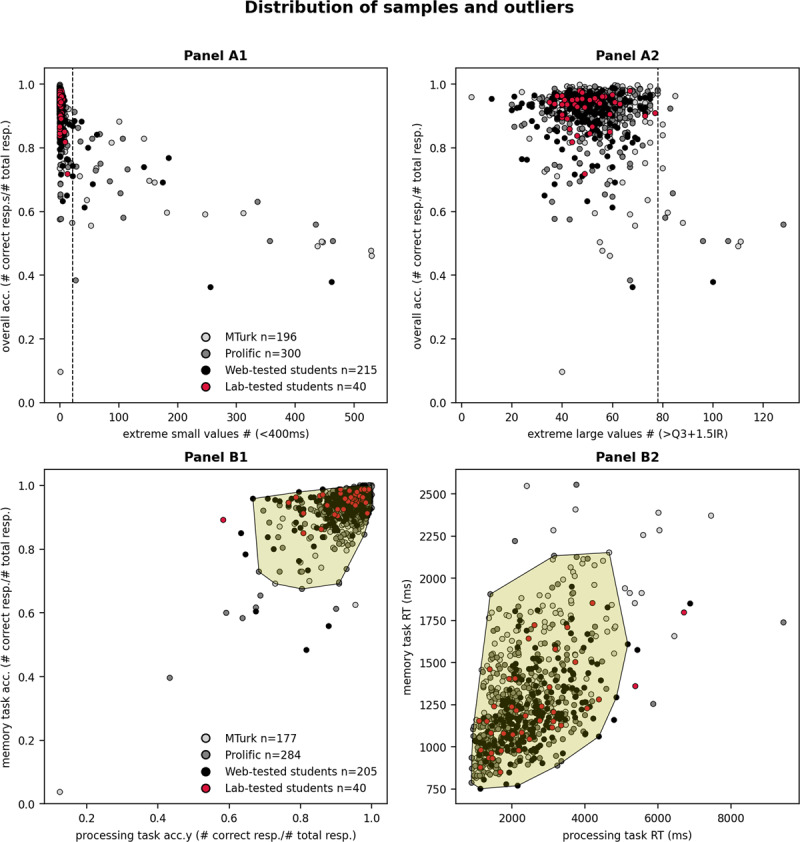
Distribution of data patterns with indication of anomalous data patterns in data from MTurk, Prolific, web-tested students and lab-tested students. *Note*: Panel A1 x-axis contains the number of extremely small values for each data pattern (< 400 ms) and Panel A2 x-axis contains the number of extremely large values (>Q3 + 1.5IR) for each data pattern. Accuracy for all trials is plotted on the y-axis. The participant pool and testing modality of each data pattern is indicated by the colour according to the legend. Our clustering method identified anomalous patterns which had many extreme values as indicated by the dashed lines. Panel B1 x-axis contains the median processing accuracy, and the y-axis contains the median memory accuracy. Panel B2 x-axis contains the median processing RT, and the y-axis contains the median memory RT. Our clustering method identified all patterns outside of the yellow zone as outliers in terms of accuracy and RT.

The average proportion of extremely small RT values per data pattern was largest for MTurk participants; one pattern from this platform contained on average 3.9% extremely small values, i.e., about 19 extremely small RTs out of 480 RTs collected from every participant. This was followed by Prolific (1.8%), web-tested students (1.6%), and lab-tested students (0.4%). The average proportion of extremely large values was also largest for MTurk participants; one pattern from this platform contained on average 10% of 480 RTs that corresponded to extremely large values. This was closely followed by Prolific participants (9.6%), web-tested students (8.9%), and lab-tested students (8.9%). Figure 2 panels A1 and A2 show that data patterns with many extreme values had low overall accuracy (i.e., low proportion correct for all responses, as illustrated on the y-axis).

We applied clustering (see Supplemental File 2) on the number of extremely small and large values to separate anomalous data patterns on each of these dimensions. No anomalous patterns were present in lab-tested students, and the presence of anomalous patterns was highest for MTurk participants (9.7% of the initial 196 participant patterns), followed by Prolific participants (5.3% of the initial 300 participant patterns), and then web-tested students (4.7% of the initial 215 participant patterns). We removed these data patterns before examining patterns that were anomalous concerning overall accuracy scores (Figure 2 Panel B1) and overall median RTs across regular memory and processing trials (Figure 2 Panel B2). Our clustering analysis revealed anomalous data patterns for average accuracies and median RTs for MTurk participants (7.1% of the initial 196 patterns), Prolific participants (3.3% of the initial 300 patterns), web-tested students (3.7% of the initial 215 patterns), and lab-tested students (7.5% of the initial 40 patterns). These anomalous patterns had particularly low average accuracy scores or particularly long median RTs.

Finally, we removed an additional small number of anomalous patterns that were outliers in terms of accuracy on verbal disruption trials (lower than .425, see Supplemental File 3), since we had only considered accuracy on regular trials in the previous analysis. Such patterns were rare across participant pools (0.5% for MTurk samples, 0.7% for Prolific, 0% for both web-tested students and lab-tested students).

When we combined all anomalous data patterns, differences between the participant pools appeared (see Table 2 second row). Fisher exact tests indicated that the proportion of anomalous patterns in web-tested students (8.4%) was not significantly higher than in lab-tested students (7.5%). Similarly, the proportion of anomalous patterns in Prolific participants (9.3%) was not significantly higher than in web-tested students. However, MTurk participants yielded significantly more anomalous patterns than Prolific participants did (17.3%, *p* < 0.05), and by consequence more than any other pool. Thus, the analysis on anomalous data patterns showed no significant effect of testing modality, but there was a significant effect of participant pool, with MTurk data indicating relatively worse quality.

### 2. Verbal disruption benchmark

**Figure 3:**
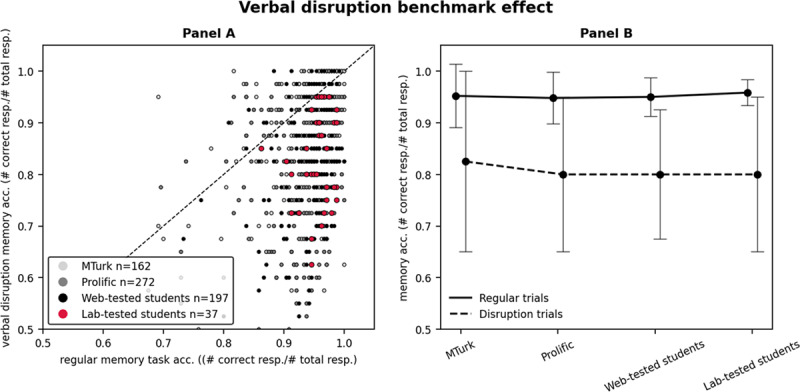
Verbal disruption benchmark effect. *Note*: Panel A x-axis indicates the regular memory accuracy for each data pattern. The y-axis indicates the verbal disruption memory accuracy. Patterns below the diagonal line present the verbal disruption benchmark effect. Panel B contains the participant pools on the x-axis. The black lines show median memory accuracy on regular and verbal disruption trials, for patterns that showed this effect. The error bars are the interquartile range, to give an indication of the middle range of performance for regular and verbal disruption trials.

Following removal of anomalous data patterns, the distribution of the remaining patterns concerning accuracy on regular memory and verbal disruption trials is displayed in Figure 3, Panel A. The diagonal line splits the data patterns that show the verbal disruption effect from those that do not show it; patterns below the diagonal line show the verbal disruption effect. All the remaining lab-tested student data patterns (100%) fall below this line, followed by web-tested students (83.8%), Prolific participants (83.1%), and lastly MTurk participants (58%). Fisher exact tests indicated that the proportion of patterns presenting the verbal disruption effect was lower for web-tested than for lab-tested students (*p* < 0.05); it was not statistically different between web-tested students and Prolific participants, but was lower for MTurk than for Prolific participants (*p* < 0.001). The combined fact that, through close monitoring we are certain that lab-tested students complied with instructions, and that all lab-tested students showed the verbal disruption effect, corroborates that the absence of this effect can be taken as evidence for task non-compliance. Figure 3, Panel B gives an indication of the size of the verbal disruption effect in those data patterns for which it was present.

In summary, this second analysis on the verbal disruption effect shows there was a significant effect of testing modality on data quality. Web-tested students replicated the verbal disruption effect less often than lab-tested students, demonstrating evidence of non-compliance in web-testing. Moreover, there was also a significant effect of participant pool, with MTurk participants replicating the verbal disruption effect less often than web-tested students and Prolific participants did, indicating further evidence of non-compliance in MTurk data (see Table 2, second row for an overview of the proportion of data patterns that did not show the verbal disruption effect in each participant pool).

### 3. RT rehearsal primacy effect

Prior to conducting the analysis on the rehearsal primacy effect, we removed all anomalous data patterns, as well as all patterns that did not show the verbal disruption effect. We analyzed the presence of a primacy effect for the remaining patterns by evaluating whether the median RT for the first presented item of a memory series was shorter than the average median RT for subsequent items in a memory series. Fisher exact tests indicated that the proportion of data patterns that showed this effect was not statistically different between Prolific participants (94.7%), web-test students (94.5%), and lab-tested students (94.6%), but MTurk participants showed the effect in a significantly fewer proportion of the remaining patterns (72.3%, *p* < 0.001). Thus, there was no significant effect of testing modality for this third analysis on the RT rehearsal primacy effect. However, there was a significant effect of participant pool as in the second analysis, with MTurk data replicating the rehearsal primacy effect to a lesser extent.

**Figure 4:**
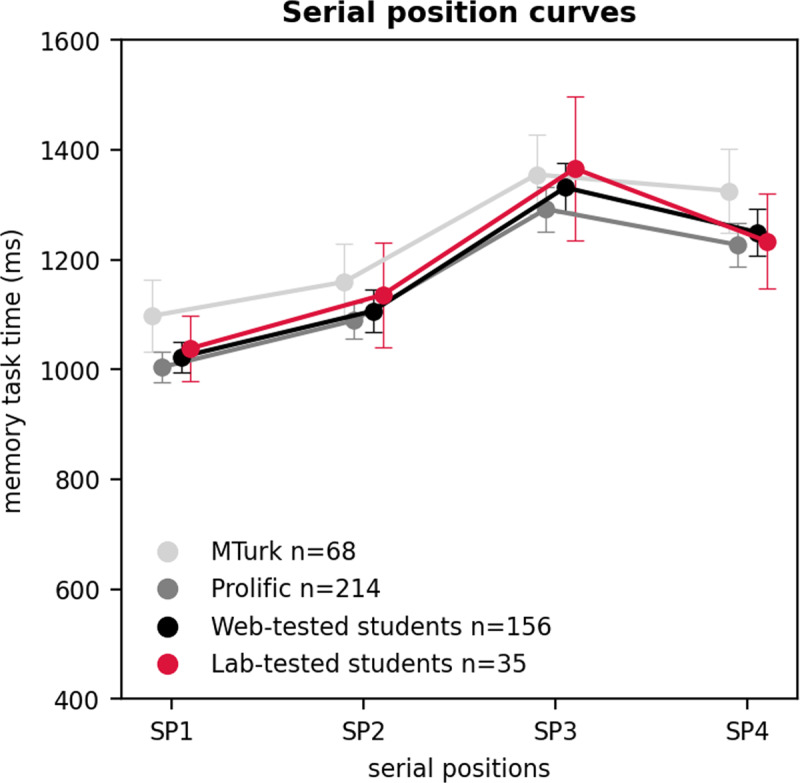
The serial position curves in MTurk participants, Prolific participants, web-tested students, and lab-tested students. *Note*: The serial position curves are calculated on the remaining data patterns that showed a primacy effect (median RT first presented letter memory series < average median RT other letters). On the x-axis we plotted the serial positions (SP), which correspond to the place that a memory probe had in the memory list. On the y-axis we plotted the average of the median memory RT of all patterns for each participant pool. The pattern medians were calculated using trials where the probe was present in the memory list, and for which participants correctly responded. Error bars represent a 95% confidence interval around the mean.

Figure 4 illustrates the serial position curves for the remaining data patterns that showed a rehearsal primacy effect. Although the shape of the serial position curve is similar between participant pools, the serial position curve appears visually less curved in the MTurk pool.

## Discussion

The novelty of our study was to assess the data quality of web-based cognitive psychology research while disentangling effects of testing modalities and participant pools. We evaluated the testing modality effect by comparing data collected in a closely monitored, typical lab-setting to data collected via unmonitored, web-testing, both from undergraduate students at the University of Geneva. We evaluated the participant pool effect by comparing data between different participants pools that were tested online, including web-tested students, MTurk and Prolific participants. We developed a working memory paradigm that is fairly standard and general in the research field. The collection of RTs and the replication of RT benchmark effects is particularly relevant for web-testing in working memory research, which is why we were interested in these assessments. Moreover, our paradigm was able to yield clear working benchmark effects (Oberauer et al., 2018) which, in our case, are particularly indicative of participants’ task compliance.

The findings of our study are summarized in Table 3. First, data quality for lab-tested students was very high overall. These data presented few extreme values or anomalous characteristics, and yielded clear and large benchmark effects across most participants, with 87.6% (95% CI [73.9, 94.5]) of patterns passing all our quality criteria. These results constitute the standard against which to compare other testing modalities and participant pools. Second, our results showed minor effects of testing modality. Some data quality is lost when conducting unmonitored web-testing of students (see Table 2, percent no verbal disruption effect). Among the web-tested student patterns, 72.6% (95% CI [66.2, 78.1]) passed all our quality criteria, corresponding to a relative loss of 17.1% compared to monitored lab-testing of students. Third, our results showed that data from crowdsourcing platforms have the potential to closely mimic data from web-tested students. Prolific data were strikingly similar to web-tested student data; we removed similar numbers of anomalous patterns, and benchmark effects were present to the same extent in both pools. Among the Prolific data patterns, 71.3% (95% CI [66.0, 76.2]) passed all our criteria. Therefore, we found that data collection via a crowdsourcing platform can be equivalent to web-testing students. This finding is quite encouraging, given that we had no way to enforce the demanding verbal disruption instruction, yet evidence for non-compliance in Prolific participants was similar to that of web-tested students, and remained acceptably low in both cases.

**Table 3:** Summary of testing modality and participant pool effects.


PARTICIPANT POOL	ANOMALOUS PATTERNS	VERBAL DISRUPTION BENCHMARK	REHEARSAL PRIMACY BENCHMARK

Lab-tested students	++	++	++

Web-tested students	++	+–	++

Prolific	++	+–	++

MTurk	––	––	––


*Note*: Best data quality is indicated by ‘++’, worst data quality is indicated by ‘– –’, and when the observations are in-between, this is indicated by ‘+–’.

Finally, our results revealed that it is crucial to select crowdsourcing participant pools carefully. MTurk participants scored consistently worse across all our quality criteria. Compared to the other participant pools, there was a larger number of anomalous patterns and fewer patterns were indicative of both the verbal disruption and rehearsal primacy effects. Only 34.7% (95% CI [28.4, 41.6]) of MTurk patterns passed all our quality criteria which is significantly less than the other samples (*p* < .001).

We do not know what factors contributed to low data quality in the participant pools, specifically on MTurk, and identifying the reasons is beyond the scope of our paper. We can, however, exclude some possibilities and present some potential avenues that may apply to our study. Compared to other participant pools, the MTurk participant pool showed a higher prevalence of extremely fast responses associated to inaccurate answers. This may indicate that response keys were pressed as fast as possible in order to complete the task faster without engaging in the task. Moreover, the MTurk participants also showed more evidence for non-compliance with instructions.

It is a limitation of our study that we do not have access to specific participant characteristics on MTurk to examine which participant characteristics may be linked to low data quality. However, our supplementary analysis on the characteristics of Prolific participants – data which we did have access to – did not show any links between demographic characteristics and data quality (see Supplemental File 4). We only found an effect of prior participant approval rating, which was positively associated to passing our quality criteria. This analysis underscores the importance of pre-screening participants by approval rating, even on Prolific where data quality may be further improved by pre-screening.

Contrary to Prolific, the importance of pre-screening by approval rating has been repeatedly stressed for MTurk. MTurk requesters or researchers should only select participants with a minimum 95% approval rating (e.g., Chandler et al., 2019; Peer et al. 2014) and with minimum 100 approved tasks (note that with less than 100 approved tasks, the approval rate is automatically set to 100%). In our study, implementing this step was not sufficient to obtain adequate data quality, as reflected by low benchmark scores and anomalies in data distributions. Participant screening can also be implemented during the task. MTurk requesters often implement attention or comprehension checks (Hauser & Schwarz, 2016) to weed out participants who do not understand nor pay attention to task instructions. In our study, implementing an instructions tutorial with performance tracking – and only accepting participants who reached a performance criterion – was also not sufficient to obtain adequate data quality.

A final possible reason for inadequate data quality, particularly from MTurk, may be unfair payment (e.g., Lovett et al., 2018, Casey et al., 2017). It should be noted, however, that our study offered compensation considered more than fair by MTurk participants themselves (see https://turkerview.com/requesters/A21LU028LEOI1T). In sum, despite our precautions, we were not able to obtain adequate data quality while using the MTurk platform for our experiment.

## Conclusion

To our knowledge, this is the first study to disentangle the effects of *testing modality* (i.e., how you test) and *participant pool* (i.e., who you test) on data quality in behavioral research. We examined data quality in the domain of cognitive psychology using a fairly typical paradigm, by way of an experimental task with precise task instructions, as well as accuracy and RT measurements.

Concerning *how you test*, unmonitored web-testing incurred minor loss of data quality compared to monitored lab-testing, perhaps unsurprisingly so. Based on our results, to err on the side of caution, recruiting 20% more participants should allow for the same quantity of high-quality data patterns as lab-testing would. However, this data quality loss is offset by the convenience of efficient parallel testing, which has a lower cost than lab-testing. Therefore, we would argue that our results encourage the use of web-testing in cognitive psychology, even with complex paradigms.

Nevertheless, our results reveal that *who you test* is of utmost importance. Prolific results were almost indistinguishable from web-tested students, but MTurk results differed very much from the other participant pools. This finding stresses the importance of carefully considering the selection of participants from crowdsourced participant pools. At the same time, this careful selection should not entail selecting only participants with very specific characteristics via strict pre-screening criteria; it is important to promote diversity in study participants. In our study, we purposefully did not exclude participants based on any criteria other than the approval rate on MTurk, and we strongly believe that including diverse samples is possible without sacrificing data quality. The results from Prolific are promising in this regard since we did not need to use any screening criteria in order to obtain acceptable data quality. Unfortunately, employing often-cited screening techniques on MTurk did not guarantee adequate data quality – at least in the present study – and it seems that on this platform additional precautions need to be taken. Our findings entail important repercussions for conducting research where many researchers may increasingly take on web-based testing, especially post-pandemic.

## Data Accessibility Statements

The data used in this research are part of a large-scale recruitment effort within our lab to collect data using a web-based behavioral paradigm, with the goal to train machine learning classification algorithms on working memory tasks. This endeavor included other conditions manipulated within the same memory task where we altered the instructions regarding how we asked participants to maintain information. For example, some participants had to continuously rehearse only the last letter of the series. These other conditions are relevant in the context of our research on working memory classification tasks. However, we did not analyze the data from these conditions in the present study, as we estimated that these data did not provide important contributions on the issue of data quality, which was our primary concern.

All data and materials are available at https://osf.io/yznm2/?view_only=3f19ab8982cc412cba388405135870da.

The experiment can be tested at the following link: (https://workthatmemory.unige.ch/mturk/). The code is not publicly available. Interested parties can contact the corresponding author.

## Additional Files

The additional files for this article can be found as follows:

10.5334/joc.259.s1 Supplemental File 1. *MTurk and Prolific participant pools.* This supplemental file contains a table with characteristics of MTurk and Prolific participant pools.

10.5334/joc.259.s2 Supplemental File 2. *Histogram of reaction times and evolution of reaction times and accuracy across trials.* This supplemental file contains a histogram of reaction times, and a justification for choosing a fast response cut-off value of 400 ms. In addition, the file also contains an investigation of potential task fatigue by analysing reaction times and accuracy across trials.

10.5334/joc.259.s3 Supplemental File 3. *Details of the clustering method.* This supplemental file contains details about the clustering method that was used to detect anomalous cases, as well as parameter choices.

10.5334/joc.259.s4 Supplemental File 4. *Participant characteristics and data quality on Prolific.* This supplemental file contains a table with characteristics of Prolific participants who passed all our quality criteria and those who did not. The file also contains an analysis examining the link between these characteristics and whether the data passed all quality criteria.

## Footnotes

Audio-recordings were important since a part of our task asked participants to say things aloud.
