# Supplementary material for: From Lab-Testing to Web-Testing in Cognitive Research: Who You Test is More Important than how You Test
Source: J Cogn. 2023 Jan 19;6(1):13. doi: 10.5334/joc.259 (PMC9854315; doi:10.5334/joc.259)
Supplement: Supplemental File 1. — MTurk and Prolific participant pools. This supplemental file contains a table with characteristics of MTurk and Prolific participant pools. [file joc-6-1-259-s1.pdf]

Characteristics of participant pools on MTurk and Prolific.

Variable	MTurk	Prolific
Size of active participant pool	~200k-300k	~200k-300k
Age		
18-29	30%	68%
30-59	64%	30%
60+	6%	2%
Gender (females)	57%	68%
Education: College degree	65%	49%
Nationality	60-90% US residents	41% US
	5-30% India	21% UK
		11% EU other
Income		
<10k	6%	11%
10k-30k	18%	18%
30k-60k	33%	26%
60k-100k	26%	24%
>100k	17%	21%
Experience		
0-100 participations	35%	80%
100-1000 participations	37%	19%
>1000 participations	28%	1%

Note. Whereas the reported values were available on Prolific by using the audience checker feature (https://app.prolific.co/audience-checker), MTurk does not directly provide such information. The reported values for MTurk are derived from Difallah, Filatova and Ipeirotis (2018, see https://demographics.mturk-tracker.com/#/countries/all for a continuously updated tracker) and by Moss et al., 2021 (see also https://www.cloudresearch.com/resources/blog/who-uses-amazon-mturk-2020-demographics/). These authors only collected information from participants who partook in their tasks, which likely introduced a bias in the representation of the participant pool.

In our own interpretation of the values in this table, what varies the most between participant pools is experience. MTurk participants have typically completed a much greater number of tasks than Prolific participants and are therefore likely much more experienced than both Prolific participants and university students (e.g., Hauser et al., 2019). It is possible that experience influences data quality, but this is not directly tested in the current study.
