# Supplementary material for: From Lab-Testing to Web-Testing in Cognitive Research: Who You Test is More Important than how You Test
Source: J Cogn. 2023 Jan 19;6(1):13. doi: 10.5334/joc.259 (PMC9854315; doi:10.5334/joc.259)
Supplement: Supplemental File 2. — Histogram of reaction times and evolution of reaction times and accuracy across trials. This supplemental file contains a histogram of reaction times, and a justification for choosing a fast response cut-off value of 400 ms. In addition, the file also contains an investigation of potential task fatigue by analysing reaction times and accuracy across trials. [file joc-6-1-259-s2.pdf]

Histogram of reaction times

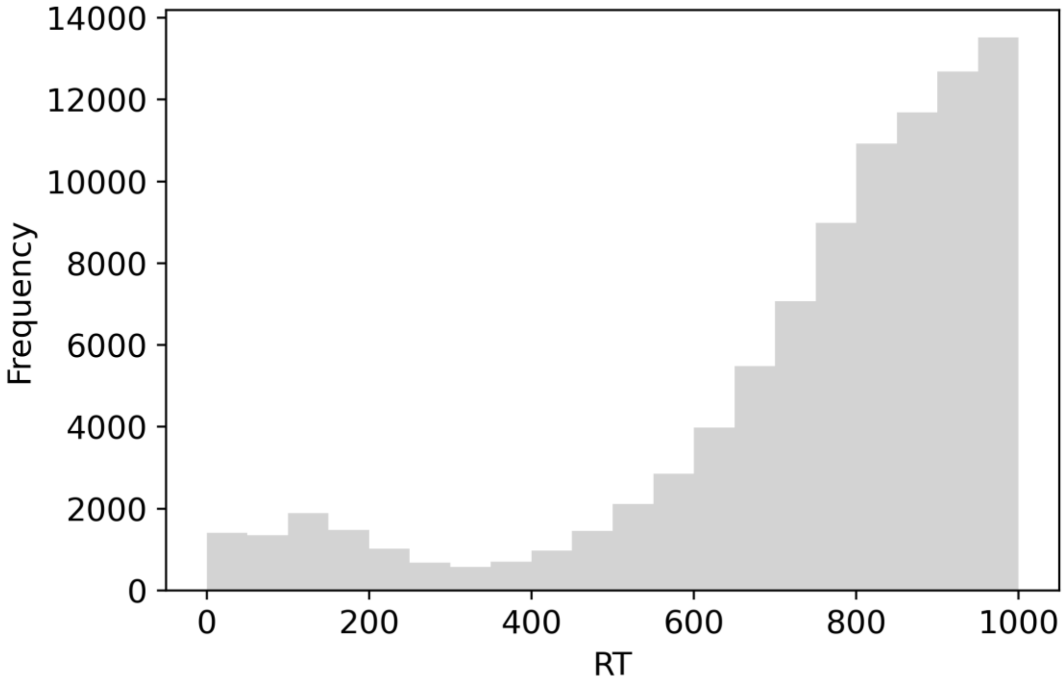

Figure 1: On the x-axis we find all RTs in range 0-1000 ms collected during the task, across participants and platforms, and including all types of responses. On the y-axis we find the frequency of RTs.

On the left of Figure 1, we can observe an anomalous distribution of RTs around 100-150 ms. These low values may reflect erratic keypresses or technical errors but may also reflect intentional behavior to bring the task to an end as fast as possible. If the data sample of a participant shows many such values, we considered their response pattern to be anomalous. The anomalous distribution overlaps with the left side of a seemingly classic RT distribution between 300 and 400 ms. Given that our goal is to detect anomalous response patterns, it is reasonable to take into consideration all values below 400 ms, and to use this as the cut-off value for determining extremely small values. Note that this choice of cut-off fast response times has no bearing on the further study of benchmark effects, since we used median values in all reported analyses.
