# Supplementary material for: From Lab-Testing to Web-Testing in Cognitive Research: Who You Test is More Important than how You Test
Source: J Cogn. 2023 Jan 19;6(1):13. doi: 10.5334/joc.259 (PMC9854315; doi:10.5334/joc.259)
Supplement: Supplemental File 3. — Details of the clustering method. This supplemental file contains details about the clustering method that was used to detect anomalous cases, as well as parameter choices. [file joc-6-1-259-s3.pdf]

Details of the clustering method

The clustering method that we used to separate anomalous participant samples was the DBSCAN algorithm (Density Based Spatial Clustering of Applications with Noise), implemented by the scikit-learn library in Python (Euclidean distance metric by default, Pedregosa et al., 2011). This implementation requires the user to choose two parameters, defining the neighbourhood region around each point (eps), and the minimum number of points required to form a dense region (min points). When there are not enough points in the neighbourhood of a particular point, then this point can be considered an outlier. The values for eps and min points are arbitrarily chosen by the user. First, we standardized the variables. Then, we used best practice heuristics to choose values of min points and eps (e.g., Rahmah & Sitanggang, 2016). We selected min points values of 4 when we applied DBSCAN on one dimension, and values of 8 when we applied it on two dimensions. For each data point, we then plotted the average distance between this point and its nearest neighbours (the number of nearest neighbours is equal to min points), from smallest to largest. Reasonable values for eps are in the elbow of this plot, at the point where the increase in average distances shows an inflection point (e.g., Rahmah & Sitanggang, 2016). This method led to the selection of different parameters for different applications of the clustering method in the detection of anomalous samples:

- Clustering on the number of extremely small values: eps = 0.05, min points = 4
- Clustering on the number of extremely large values: eps = 0.1, min points = 4
- Clustering on the accuracy of the processing and memory tasks: eps = 1, min points = 8
- Clustering on the RT of the processing and the memory tasks: eps = 0.5, min points = 8
- Clustering on verbal disruption trial accuracy: eps = 0.25, min points = 4
