# Supplementary material for: From Lab-Testing to Web-Testing in Cognitive Research: Who You Test is More Important than how You Test
Source: J Cogn. 2023 Jan 19;6(1):13. doi: 10.5334/joc.259 (PMC9854315; doi:10.5334/joc.259)
Supplement: Supplemental File 4. — Participant characteristics and data quality on Prolific. This supplemental file contains a table with characteristics of Prolific participants who passed all our quality criteria and those who did not. The file also contains an analysis examining the link between these characteristics and whether the data passed all quality criteria. [file joc-6-1-259-s4.pdf]

Participant characteristics and data quality on Prolific.

We retrieved participant characteristics (i.e., gender, age, country of residence, time taken to do the task, number of tasks completed, approval rate) from the Prolific participants, which can be consulted in Table 1. Note that collecting these data was not part of our planned study, but that participants on Prolific consented to provide these data to researchers as part of the platform policy. The data of 6 participants are missing because they did not correctly report session codes, so we were not able to retrieve their characteristics. Note that it was notpossible to obtain participant characteristics from MTurk, without having intentionally requested this information as part of the experiment. Furthermore, we also did not request this information from lab-tested and web-tested students. These data allowed us to explore whether any specific participant characteristics were linked to data quality, by comparing characteristics of participants who yielded data patterns that met our quality criteria (N = 209) and characteristics of participants who did not yield such data patterns (N = 85).

Table 1. Characteristics of Prolific participants who pass our quality checks, and participants who fail one or more checks.

Variable	Prolific pass		Prolific fail	
Gender	Female	36.5%	Female	42.0%
	Male	63.5%	Male	58.0%
Age	<22	25%	<23	25%
	22-35	50%	23-42	50%
	>35	25%	>42	25%
Country of residence[a]	UK	31.1%	UK	34.1%
	Poland	17.0%	Poland	12.2%
	Portugal	8.7%	Portugal	7.3%
	US	6.8%	US	13.4%
	Greece-Italy	11.7%	Mexico	6.1%
Time taken (min)	<37	25%	<35	25%
	37-51	50%	35-59	50%
	>51	25%	>59	25%
Tasks done	<76	25%	<80	25%
	76-334	50%	80-310	50%
	>334	25%	>310	25%
Approval rate	<98.8%	25%	<98.1%	25%
	98.8-100%	75%	98.1-99.7%	50%
			>99.7%	25%

[a]We report the 5 most prevalent countries of residence.

With logistic regression we assessed the effects of participant characteristics on the likelihood that the data of a participant would meet all our criteria. The logistic regression model was statistically significant, $X^2 = 15.06$, $p = .01$. The model explained 7.4% (Nagelkerke R^2) of the variance and correctly predicted 98.5% of cases that met our criteria, but only 7.4% of cases that did not meet our criteria. Lower Prolific approval rating was the only participant characteristic associated to a lower probability of meeting our criteria, $p = .001$. The other participant characteristics did not have any statistically significant effect (*p*s > 0.2). From this analysis and from the table, it appears that there is no association between different demographic characteristics (e.g., age, gender, country) and failing to meet the quality criteria.
